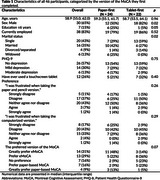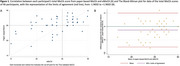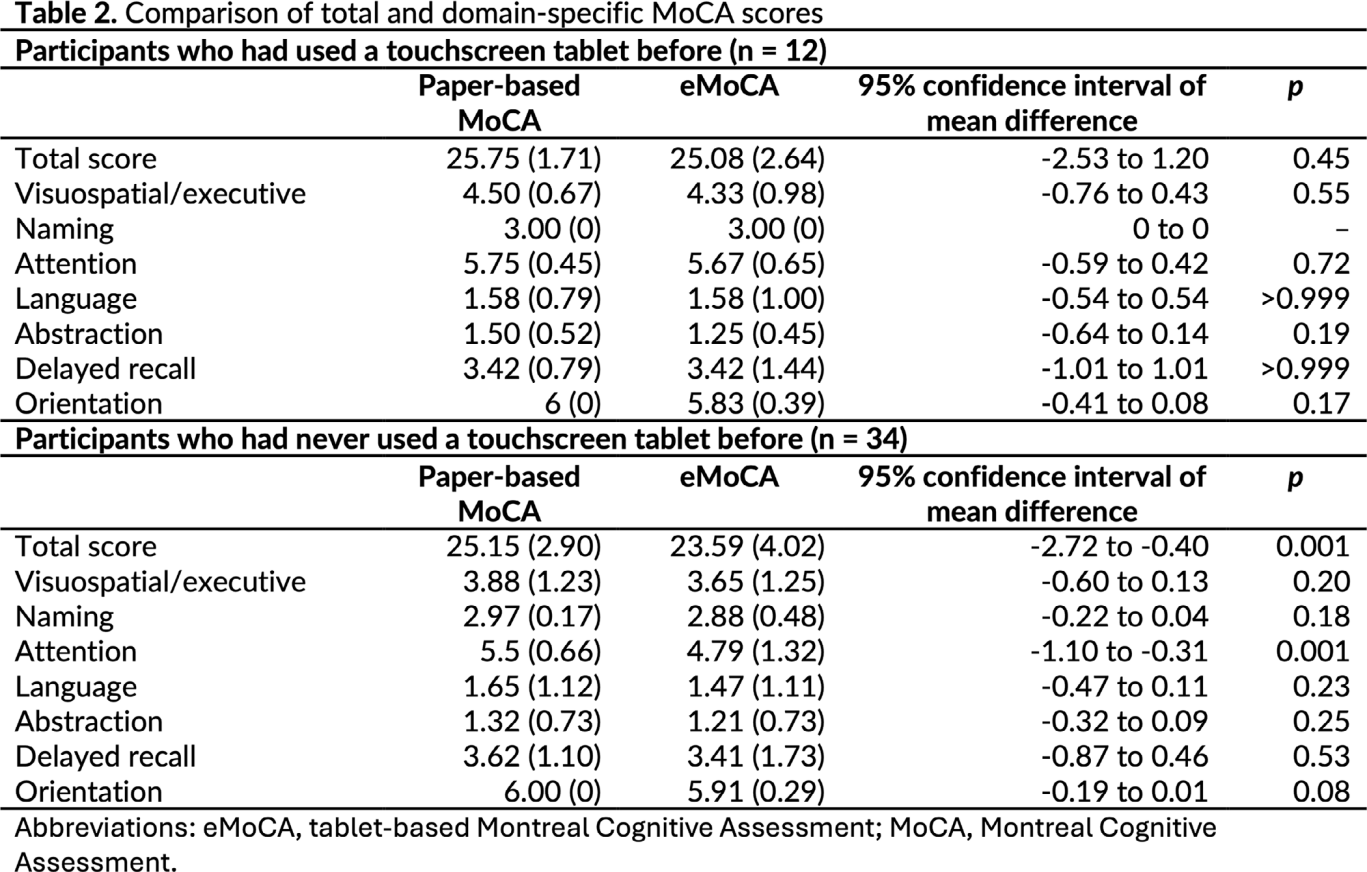# The Validity of Computerized Montreal Cognitive Assessment among Aging People Living with HIV

**DOI:** 10.1002/alz70860_107653

**Published:** 2025-12-23

**Authors:** Akarin Hiransuthikul, Thanapoom Taweephol, Netchanok Timachai, Saowaluk Suksawek, Chuleeporn Wongvoranet, Tanakorn Apornpong, Kittithatch Booncharoen, Solaphat Hemrungrojn, Anchale Avihingsanon

**Affiliations:** ^1^ HIV‐NAT, Thai Red Cross AIDS Research Centre, Bangkok, Thailand; ^2^ Department of Preventive and Social Medicine, Faculty of Medicine, Chulalongkorn University, Bangkok, Thailand; ^3^ Department of Microbiology, Faculty of Medicine, Chulalongkorn University, Bangkok, Thailand; ^4^ Neurocognitive Unit, Division of Neurology, Department of Medicine, Faculty of Medicine, Chulalongkorn University, Bangkok, Thailand; ^5^ Department of Psychiatry, Faculty of Medicine, Chulalongkorn University, Bangkok, Thailand; ^6^ Center of Excellence in Tuberculosis, Department of Medicine, Faculty of Medicine, Chulalongkorn University, Bangkok, Thailand

## Abstract

**Background:**

Cognitive assessment plays a crucial role as the initial step in cognitive care. We aimed to determine the validity between a traditional paper‐based and tablet‐based cognitive assessment tool among aging Thai people living with HIV (PWH).

**Method:**

PWH aged ≥50 years underwent cognitive assessment using the Thai‐validated Montreal Cognitive Assessment (MoCA). Participants were randomly assigned to receive either the paper‐based MoCA or the tablet‐based MoCA (eMoCA) first. Two weeks later, participants returned to complete the alternate version of the MoCA. Pearson correlation was used to determine the strength of the relationship between the paper‐based MoCA and the eMoCA scores. Concordance correlation coefficients (CCC) were calculated, and a Bland‐Altman plot was employed to determine the level of agreement between the two testing methods.

**Result:**

Among 46 participants included in the analysis, 12 (26.1%) had experience using a touchscreen tablet (Table 1). The score discrepancy between the two MoCA versions ranged from ‐8 to 6, with a mean (SD) difference of ‐1.33 (3.22). The Pearson correlation coefficient between the paper‐based MoCA and the eMoCA was r = 0.54 (*p* = 0.001) (Figure 1A), with a concordance correlation coefficient of 0.47. The Bland‐Altman plot showed 95% limits of agreement between ‐7.63 and 4.98 (Figure 1B). Among participants with prior touchscreen tablet experience, scores between the paper‐based MoCA and the eMoCA were comparable. However, those without prior touchscreen experience had significantly lower scores on the eMoCA compared to the paper‐based MoCA (mean difference ‐1.56, 95% CI ‐2.72 to ‐0.40) (Table 2).

**Conclusion:**

The eMoCA demonstrated moderate performance compared to the paper‐based MoCA, with prior touchscreen tablet experience significantly affecting the validity of the MoCA scores between the two versions. Clinicians should consider individuals’ level of touchscreen experience before selecting the administration modality.